# Fast X-ray microdiffraction techniques for studying irreversible transformations in materials

**DOI:** 10.1107/S0909049511002640

**Published:** 2011-03-16

**Authors:** Stephen T. Kelly, Jonathan C. Trenkle, Lucas J. Koerner, Sara C. Barron, Nöel Walker, Philippe O. Pouliquen, Mark W. Tate, Sol M. Gruner, Eric M. Dufresne, Timothy P. Weihs, Todd C. Hufnagel

**Affiliations:** a Department of Materials Science and Engineering, Johns Hopkins University, Baltimore, MD, USA; b Department of Physics, Cornell University, Ithaca, NY, USA; c Department of Electrical and Computer Engineering, Johns Hopkins University, Baltimore, MD, USA; d Cornell High Energy Synchrotron Source, Ithaca, NY, USA; e Advanced Photon Source, Argonne National Laboratory, Argonne, IL, USA

**Keywords:** X-ray diffraction, phase transformations

## Abstract

A pair of techniques have been developed for performing time-resolved X-ray microdiffraction on irreversible phase transformations. In one technique capillary optics are used to focus a high-flux broad-spectrum X-ray beam to a 60 µm spot size and a fast pixel array detector is used to achieve temporal resolution of 55 µs. In the second technique the X-rays are focused with Kirkpatrick–Baez mirrors to achieve a spatial resolution better than 10 µm and a fast shutter is used to provide temporal resolution better than 20 µs while recording the diffraction pattern on a (relatively slow) X-ray CCD camera. Example data from experiments are presented where these techniques are used to study self-propagating high-temperature synthesis reactions in metal laminate foils.

## Abstract 2

Techniques are described for X-ray diffraction combining micrometer-scale spatial resolution with microsecond-scale temporal resolution for studying rapid localized irreversible transformations in materials.

## 1. Introduction

Time-resolved X-ray diffraction is widely used for studying structural changes in materials. For transformations that can be reversibly excited, it is possible to exploit the pulsed nature of synchrotron radiation to achieve temporal resolution better than 1 ns (Rousse *et al.*, 2001 ▶). In this ‘pump–probe’ technique a high-speed chopper is typically used to select a single X-ray pulse, or a train of pulses with a specified interval, from the synchrotron beam. An excitation signal (such as a laser pulse) is synchronized to the time structure of the synchrotron such that the transformation of interest is excited at a pre­determined time relative to the arrival of an X-ray pulse at the specimen. The duration of an individual X-ray pulse defines the temporal resolution of the experiment which, depending on the electron bunch structure of the synchrotron, is typically of the order of 100 ps. Although the short-duration pulse may result in a poor signal-to-noise ratio for a single pulse, an acceptable signal can be built up by exciting the transformation many times.

Many transformations of interest, however, are irreversible and therefore cannot be studied by a repeated pump–probe technique. In principle, one could use a similar approach to isolate a single X-ray pulse from the synchrotron bunch structure, but even with third-generation synchrotrons each pulse contains too few photons to achieve an acceptable signal-to-noise ratio. [We note that this situation is likely to change with the advent of fourth-generation light sources, such as hard X-ray free-electron lasers (Pellegrini & Stohr, 2003 ▶; Emma *et al.*, 2010 ▶).]

One can apply a pump–probe technique to irreversible transformations in a single-shot mode, using a suitably long pulse; by repeating the experiment on multiple specimens with various delays between pump and probe pulses a complete picture of the transformation sequence can be developed. This approach relies on the transformation occurring very repeatably with respect to the pump signal timing, with the uncertainty being smaller than the desired temporal resolution (width) of the probe pulse. If the temporal uncertainty is large, however, it becomes necessary to sense the transformation during the experiment, adjusting the experimental timing accordingly. In this way a signal obtained from the transformation itself controls the timing by either signaling a shutter to produce a probe pulse or a fast detector to commence data acquisition.

Therefore, a different type of time-resolved X-ray experiment operates on longer time scales and treats the X-ray beam as a continuous source of photons. In these experiments suitable temporal resolution can be achieved in one of two ways. If the transition of interest occurs on a time scale much longer than the time necessary to collect a diffraction pattern (given the intensity of the source and the sensitivity and response time of the detector), then one can simply illuminate the specimen with X-rays continuously, collecting multiple diffraction patterns over the course of the transformation in a single specimen. The high photon flux available at second- and third-generation synchrotron sources and the advent of extremely fast detectors have made measurements of this type possible with sub-millisecond temporal resolution (Barna *et al.*, 1997 ▶; Laggner *et al.*, 1989 ▶).

On the other hand, if the transformation occurs with a characteristic time faster than the response time of the detector, one can employ a shutter to produce an X-ray pulse of suitable duration relative to the rate of transformation. In such an experiment the temporal resolution is determined by the duration of the X-ray pulse produced by the shutter (from the approximately continuous X-ray beam) and not the response time of the detector. Each specimen yields only one diffraction pattern, but if the transformation is sufficiently reproducible a sequence of patterns can be collated by repeating the experiment on multiple specimens and referencing the time at which each pattern was collected to events occurring within the specimen. Measurements of this type with millisecond resolution have been made by many groups (Fuoss *et al.*, 1992 ▶; Irving & Maughan, 2000 ▶).

Additional complication arises if the transformation of interest is not only fast but spatially localized. In most of the work described above the spatial resolution of the experiment was determined by the size of the X-ray beam (typically 0.25–1 mm). If the transformation occurs on a spatial scale much smaller than this, then changes in the X-ray scattering owing to the transformation may be swamped by scattering from the surrounding material. One way to achieve the necessary spatial resolution is to produce an X-ray beam similar in size to the region undergoing the transformation. The easiest way to do this is to use an aperture (slits), but this comes at the cost of reduced flux which, in turn, limits the signal-to-noise ratio and possibly the temporal resolution of the experiment. Alternatively one can focus the X-ray beam; the resulting improvement in flux, and thus temporal resolution, may be worth the additional complexity in the experiment.

**Figure 1:**
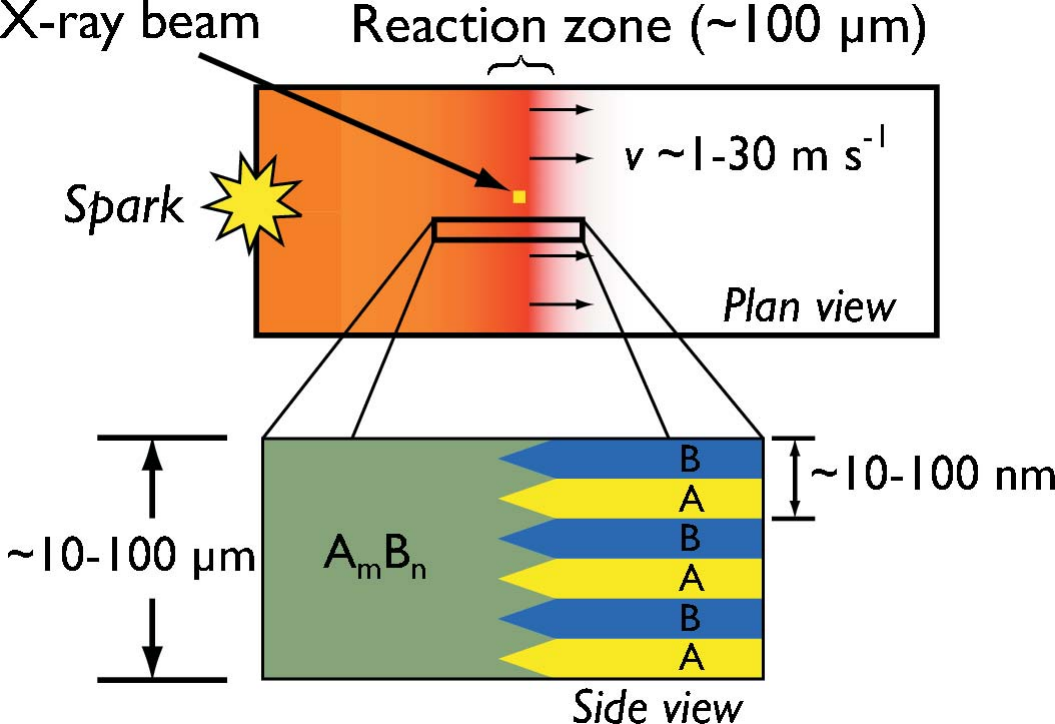
Schematic of the self-propagating reactions in metal multilayer foils, which are the focus of this work. The foils transform from initially alternating layers of two metals (represented by *A* and *B* here) to a final *A*_*m*_ *B*_*n*_ intermetallic phase as determined by the overall composition of the starting foil. In order to deduce the proper phase formation sequence in the reactions we collect time-resolved diffraction patterns as the reaction front crosses the (fixed) location of a focused X-ray beam while keeping the exposure/collection time short enough that the beam remains within the reaction zone during the measurement. The beam sizes in our experiments ranged from 60 µm using capillary optics at CHESS to 7 µm using Kirkpatrick–Baez focusing mirrors at APS.

In this paper we describe a pair of recent X-ray diffraction experiments with temporal and spatial resolution around 15–50 µs and 10–50 µm, respectively. Our transformations of interest are self-propagating reactions in nanoscale metallic multilayers (Rogachev, 2008 ▶). In these reactions a narrow (∼100 µm) reaction front propagates across the multilayer foil at speeds of ∼1–10 m s^−1^, and can heat the foil to over 1773 K in under 100 µs (Weihs, 1998 ▶). Fig. 1 ▶ shows a schematic of a representative reaction in a foil specimen. The width and velocity of the reaction front couple to determine the spatial and temporal resolution necessary to successfully probe these reactions and collect diffraction patterns from within the reaction front itself (described in detail in §2). These reactions are similar to self-propagating reactions in powder compacts, which have been previously investigated using diffraction techniques with millisecond temporal resolution (Larson *et al.*, 1991 ▶; Wong *et al.*, 1990 ▶; Stephenson *et al.*, 1989 ▶).

The reaction front velocities in these foils are fairly consistent, but slight differences from specimen to specimen result in variations of ∼5–10%. There is additional uncertainty in the ignition of the reaction, which is typically of the order of ±100 µs using our electrical ignition set-up (described in §4.4). Therefore, the added control of the techniques described here (using a fast shutter or fast detector) over a traditional pump–probe approach becomes significant when probing the reactions at time scales finer than ∼0.1–1 ms, depending on the exact reaction velocity.

In the first experiment we describe, conducted at the Cornell High Energy Synchrotron Source (CHESS), we achieved the necessary temporal resolution with a fast detector and the necessary spatial resolution by focusing the X-rays with capillary optics. In the second experiment, conducted at the Advanced Photon Source (APS), we used a fast shutter to produce single X-ray pulses with the necessary temporal resolution, and focused the X-ray beam with Kirkpatrick–Baez mirrors. We discuss the relative advantages and disadvantages of each approach, and describe prospects for future enhancements of these techniques. While reactive multilayer foils present an exceptional opportunity to investigate localized transformations under extreme heating rates, the techniques we outline here are general and can be applied to other systems requiring similar spatial resolution or temporal resolution (or both) for X-ray scattering studies.

## 2. Description of specimens and experiment

In order to provide context for the detailed discussion that follows, we begin by briefly reviewing the experiment as a whole. The specimens are metallic multilayer foils, fabricated by DC magnetron sputter deposition onto polished brass substrates. After deposition, we removed the foils from the brass substrates to yield free-standing specimens (∼1 × 3 cm). For the experiments described in §3 the specimens were Al/Ni multilayers with a nominal overall composition of Al_3_Ni_2_ (excluding V) and a bilayer period of 100 nm with total thickness ∼30 µm. For the experiments described in §4 the specimens were Al/Zr multilayers with a nominal overall composition of Al_3_Zr and a bilayer period of 95 nm with total thickness of ∼60 µm. Additional details regarding the specimen preparation can be found elsewhere (Trenkle *et al.*, 2010 ▶). The sample thicknesses were chosen to maximize the diffracted beam intensity for each foil chemistry at the respective X-ray energy used in each set of experiments (8 keV for the Al_3_Ni_2_ foils at CHESS and 12 keV for the Al_3_Zr foils at APS).

As mentioned above, the reaction front width and velocity combine to dictate the spatial and temporal resolution necessary to collect diffraction data from within the advancing reaction front. More specifically, these variables are related by the expression *r*_min_ = *l* − *v*_rxn_Δ*t*_X-ray_ where *r*_min_ is the minimum probe size (spatial resolution), *l* is the width of the reaction front, *v*_rxn_ is the reaction velocity and Δ*t*_X-ray_ is the X-ray pulse duration (temporal resolution). As an example, this means that a foil with *l* = 100 µm and *v*_rxn_ = 3 m s^−1^ requires temporal resolution of ∼20 µs with spatial resolution better than 40 µm. On the other hand, if the reaction front is only ∼50 µm wide with the same reaction velocity the experiment requires temporal resolution around 14 µs with spatial resolution better than 10 µm. In addition to these lower bounds on the spatial and temporal resolution, upper bounds exist as well. X-ray beam sizes much smaller than the grain sizes in the foils are not advantageous as they cause the resulting diffraction patterns to display distinct spots instead of the continuous rings necessary for our phase analysis. As the X-ray beam size approaches the average grain sizes in the foil specimens (typically around 10–100 nm as deposited, with the grains growing to around 1 µm after the reaction completes), the resulting diffraction patterns display spots instead of continuous rings, making definite phase identification extremely difficult. Indeed, in our work here using beam sizes down to ∼7 µm we observe very spotty diffraction patterns from the post-reaction foils and must average the patterns from tens of foil specimens to retrieve an acceptably smooth azimuthally integrated pattern. Similarly, as the temporal resolution becomes finer and finer the number of photons contained in each X-ray pulse decreases and eventually the measurements become flux limited as a complete pattern cannot be retrieved from a single pulse.

For the X-ray diffraction experiments, we mount the foil specimens in a specially designed holder that allows us to remotely ignite the reaction in the foil while holding the sample in the X-ray beam path; this holder and the ignition system are described in detail in §4.4. After ignition, the reaction rapidly propagates as a narrow front along the length of the foil (Trenkle *et al.*, 2008 ▶). In order to determine the time at which the reaction front will cross the beam position, we use a fiber-coupled photodiode to sense the light emitted by the front as it approaches the position of the X-ray beam. As the reaction front passes the X-ray beam, we record diffraction data in one of two ways, either by recording patterns continually on a fast detector (§3) or by recording a single pattern from a pulse generated by a fast shutter at a pre-determined time relative to the arrival of the reaction front at the X-ray beam, repeating this process for multiple specimens (§4). In both cases we then assemble the collected patterns into a series showing the evolution of structures in the reaction front as a function of time.

## 3. Fast detector experiments at CHESS

**Figure 2:**
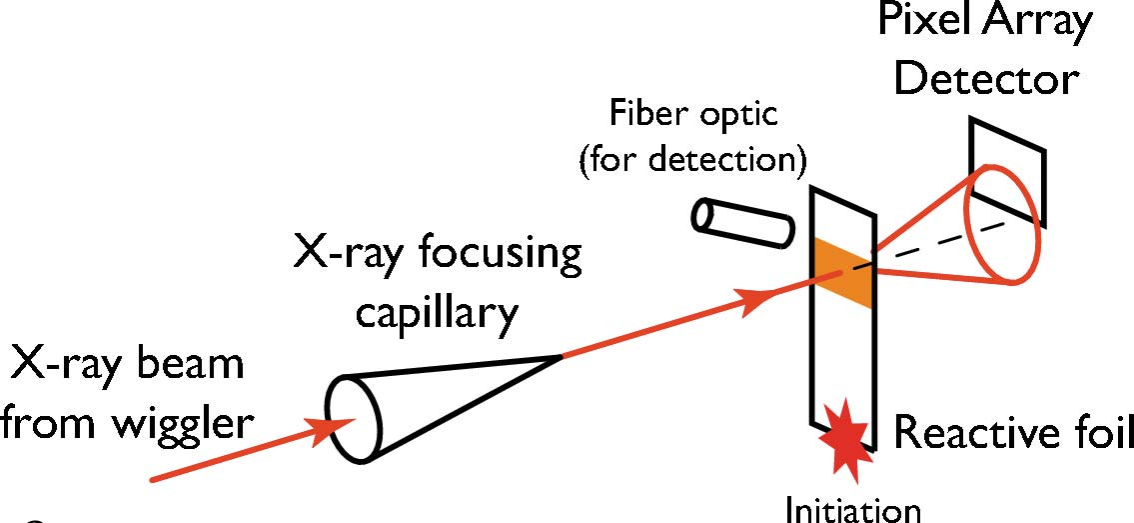
The experimental set-up for the experiments conducted with the pixel array detector (PAD) at CHESS. The monochromated X-rays pass through the glass capillary focusing optics to reduce the beam size and immediately impinge on the sample. The scattered X-rays are then collected on the PAD, with temporal resolution established by the PAD electronics. The data collection is synchronized to the foil reaction by using a fiber optic coupled to a photodiode to detect the light from the approaching reaction front and begin the data collection sequence.

Our first approach to time-resolved microdiffraction studies of self-propagating reactions used a fast pixel array detector, with the X-rays focused to a small spot by capillary optics. These experiments were conducted at CHESS, and some of our results have been presented previously (Trenkle *et al.*, 2008 ▶, 2010 ▶). Fig. 2 ▶ shows a schematic of the experiment. In these experiments we positioned the pixel array detector (PAD) above the direct beam and centered on a scattering angle of ∼45°, with the detector face roughly perpendicular to the scattered beam at this angle. In this configuration we recorded ∼15% of the diffraction rings over the range of scattering vectors from approximately 1.8 to 4 Å^−1^.

### 3.1. Temporal resolution

For the experiments at CHESS we used a pixel array detector having a pixelated silicon detection layer which is directly solder bump-bonded to a complementary metal oxide semiconductor (CMOS) electronics layer. This arrangement provides each detection pixel with its own processing electronics and enables data acquisition with sub-microsecond temporal resolution. The particular PAD used for the experiments described here had an active area of 15.0 mm × 13.8 mm (150 µm square pixels arranged in a 100 × 92 grid). PADs are described in detail elsewhere (Koerner *et al.*, 2009 ▶; Eikenberry *et al.*, 1998 ▶; Barna *et al.*, 1997 ▶; Rossi *et al.*, 1999 ▶).

**Figure 3:**
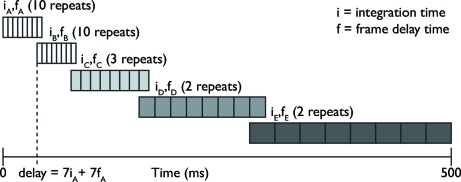
Illustration (not to scale) of the data collection sequence we used with the PAD. To cover times from 0 to 500 ms we used integration times of *i*_*A*_ = *i*_*B*_ = 50 µs, *i*_*C*_ = 500 µs and *i*_*D*_ = *i*_*E*_ = 5000 µs with interframe delay times of *f*_*A*_ = *f*_*B*_ = 5 µs, *f*_*C*_ = 500 µs, *f*_*D*_ = 5000 µs and *f*_*E*_ = 45000 µs.

Using the associated electronics for the PAD, the user can set (i) the delay between an external trigger signal and the start of data collection, (ii) the integration time for each frame, and (iii) the delay between successive frames. Since the PAD could collect eight frames for each specimen, we were able to cover a slice of the reaction spanning (8 × the integration time) plus (7 × the delay time) in each set. We spaced the delay times for each collection set such that the first frame of one set overlapped with the last frame from the adjacent set, allowing us to ensure continuity and consistency of data between sets. As we show below, the passage of a self-propagating reaction front involves events that occur on time scales ranging from microseconds to hundreds of milliseconds; although the reaction front passes the X-ray beam in ∼100 µs, heating continues to about 1 ms, and phase transformations occur during cooling through much longer times. Because of the eight frame limit, we could not collect data over the entire course of the reaction (∼500 ms) with the short integration times necessary to study the rapid initial events (<100 µs). Instead, we collected data from multiple specimens, using integration times ranging from 50 µs for the earliest portions of the reaction to 5 ms for measurements made later in the reaction sequence. In order to provide a reproducible temporal reference for the data sets, we used an optical signal from the approaching reaction as the reference point for our measurements (described in §3.2). Fig. 3 ▶ illustrates this timing scheme graphically.

To achieve a suitable signal-to-noise ratio in the data, and to ensure reproducibility, we repeated each measurement (*i.e.* each unique combination of delay and integration time) between two and ten times (more for the shortest integration times and fewer for the longest). After checking for reproducibility, the final data consisted of averages of the data sets from each measurement.

### 3.2. Timing

The timing scheme described above requires a means to initiate data collection at a controlled time relative to the moment when the reaction front passed the X-ray beam. In principle, one could initiate data collection at a defined time relative to the signal for igniting the foil, but in practice this does not work well owing to uncertainties in both the time required for ignition and the velocity of the reaction front. Instead, we detected the reaction front optically as it approached the X-ray beam position. We positioned a 200 µm-diameter optical fiber with 8.5° collection angle at 1–2 cm from the foil holder, aimed near the position of the X-ray beam on the specimen. The light collected was directed onto a photodiode connected to a pulse height analyzer (PHA). Light from the approaching reaction front caused the photodiode signal to rise and, when it reached a predetermined threshold, the PHA sent a voltage level pulse to the PAD; this time was used as a reference time (*t* = 0) for the data subsequently collected. With this technique we could predict the time the reaction front would reach the X-ray beam with an uncertainty of ±20 µs. To collect data at later times, we inserted a delay into the timing sequence (between the PHA trigger condition and sending the signal to commence data collection on the PAD) using the electronics associated with the PHA.

### 3.3. X-ray focusing with capillary optics

The experiments at CHESS were conducted on wiggler beamline A2 (Kazimirov *et al.*, 2006 ▶) using 8.2 keV X-rays. This energy is just below the Ni *K* absorption edge, minimizing absorption and avoiding background from Ni *K* fluorescence. To maximize the incident beam flux, we used a sagittally focusing W/B_4_C multilayer monochromator with 1.9% energy bandpass. The X-rays were focused with a glass capillary (PEB605), which gave us a 60 µm spot size at the sample distance of 5.5 cm (Huang & Bilderback, 2006 ▶). This arrangement yielded approximately 10^13^ photons s^−1^ in the X-ray beam at the sample.

## 4. Fast shutter experiments at APS

**Figure 4:**
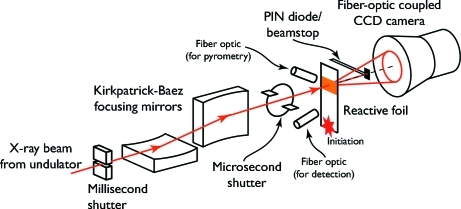
Experimental set-up for the fast shutter and CCD detector experiments performed at APS. The X-rays from the synchrotron pass first through the heat-load (millisecond) shutter, which blocks the intense X-ray beam the majority of the time to prevent damage to the focusing mirrors. From there the beam is reflected from the Kirkpatrick–Baez focusing mirrors to achieve the desired spatial resolution. The beam then passes through the fast shutter, establishing the temporal resolution of the measurement. Next, the beam hits the sample and scatters off the foil. The scattered X-rays are collected on the CCD detector, forming the diffraction pattern, and the direct beam hits the PIN diode and beamstop. At the same time, a fiber optic positioned to receive light from the reacting foil records *in situ* pyrometry data while a separate fiber optic positioned closer to the ignition point provides a signal for timing the experiment.

The key elements in the experimental set-up at APS (Fig. 4 ▶) are as follows.

(i) A slow (millisecond) shutter, the primary purpose of which is to limit the heat load on the focusing mirrors.

(ii) A pair of Kirkpatrick–Baez focusing mirrors.

(iii) A fast (microsecond) shutter, to produce the X-ray pulse for the diffraction pattern.

(iv) A Si PIN diode for timing the X-ray pulse.

(v) A pair of optical fibers pointed at the foil to collect light from the reaction front for timing and pyrometry.

(vi) A fiber-optic-coupled X-ray CCD camera; since the CCD is much slower than the PAD, the temporal resolution in this experiment is determined by the duration of the X-ray pulse.

With this method we could collect only one diffraction pattern from each specimen, compared with eight with the PAD. However, the larger size of the CCD detector relative to the PAD offered significant benefits. In particular, it allowed us to capture a wider range of scattering vector magnitudes (further aided by the higher X-ray energy used at APS) and a larger azimuthal fraction of the diffraction rings, enhancing the signal-to-noise ratio in the data and avoiding problems with powder averaging. The signal-to-noise ratio was also improved by the higher photon flux at APS.

### 4.1. Temporal resolution

**Figure 5:**
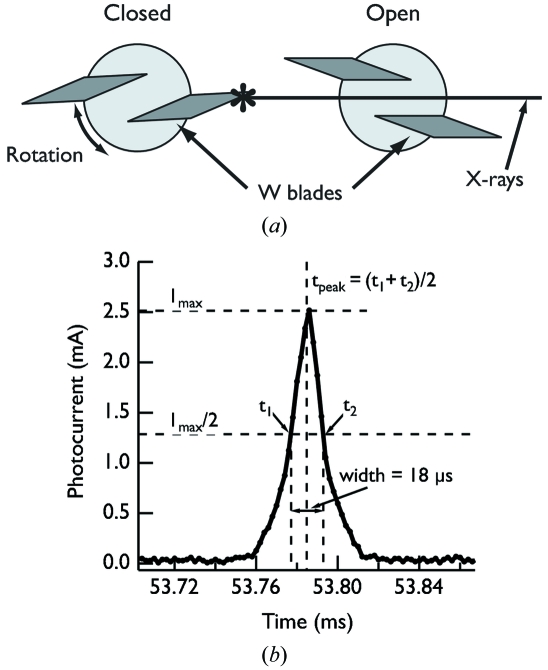
(*a*) Diagram of the shutter used in the experiments utilizing the MAR 165 CCD camera. The tungsten blades (dark gray parallelograms) attach to the galvanometer heads (light gray circles) with epoxy and fully attenuate the incident X-ray beam in the ‘closed’ position. The shutter on the left shows the position in the ‘closed’ state while the shutter on the right shows the ‘open’ state. (*b*) Example 18 µs X-ray pulse as recorded on a Si PIN diode placed downstream of the samples and the points used to determine the pulse position and width.

In the APS experiments we achieved the necessary temporal resolution by generating a short X-ray pulse with a fast shutter. This shutter needed to actuate quickly and reproducibly, fully attenuate the hard X-ray beam, and handle the heat load of the focused pink beam from the undulator. We used a shutter based on a factory-modified commercial laser-scanning galvanometer head, originally developed to shutter a large-aperture high-energy (50–100 keV) X-ray beam (Goetze & Lienert, 2009 ▶). The shutter, shown in Fig. 5(*a*) ▶, uses a small galvanometer head (Cambridge Technologies 6220H) to rotate a pair of tungsten blades along an axis perpendicular to the X-ray beam, which acts to open and close the shutter aperture. The galvanometer unit is controlled by a factory-calibrated servo driver circuit which takes in a ±10 V reference voltage signal and translates that into a ±20° rotational position at the galvanometer head. We moved the galvanometer head by rapidly switching the voltage signal (supplied by an Agilent E3620A power supply) from a low reference value (∼−6 V) to a high reference value (∼6 V) using a high-speed analog semiconductor switch (Vishay DG403B) controlled with TTL voltage level signals from a delay generator.

We utilized a two-galvanometer arrangement in the fast shutter with two independently controlled galvanometer heads placed close (∼2 cm) to each other along the X-ray beam path. Beginning from a state where one galvanometer is closed and the other is open, we generate an X-ray pulse by first opening the initially closed galvanometer and then closing the other one. By carefully changing the relative opening and closing times of the individual galvanometers we were able to adjust the X-ray pulse duration. With two galvanometers each one acts to either open or close the aperture so that the blades can eclipse the beam at maximum rotational velocity and, because the motions are independent of each other, the opening and closing times of each galvanometer can be adjusted to produce shorter pulses (by ∼10×) than would be possible with a single-galvanometer implementation. (In a single-galvanometer arrangement the galvanometer must rotate in one direction to open and then reverse the motion to close again, overcoming the inertia of the initial rotation to do so.) We positioned the fast shutter ∼4 cm upstream of the sample location, between the focusing mirrors and the samples.

A small silicon PIN diode (NXP Semiconductors BAP64-02) mounted on a printed circuit board arm and positioned in the direct beam downstream of the samples and immediately upstream of a tungsten beamstop allowed us to monitor the X-ray pulses produced by the shutter. The PIN diode, which will ultimately be part of an integrated beamstop assembly as described elsewhere (Ellis *et al.*, 2003 ▶), generated a photocurrent in the presence of the direct X-ray beam which we sent to a current–voltage preamplifier (Stanford Research Systems SR570) operating with a gain of 1 mA V^−1^. At this gain setting the 1 MHz amplifier bandwidth allowed us to resolve the X-ray pulse features on the time scales relevant to our experiment. We read the voltage output from the preamplifier on a PC-based oscilloscope (Scope4PC).

After optimizing the fast-shutter timing we were able to produce ∼18 µs-long X-ray pulses, as illustrated in Fig. 5(*b*) ▶. We controlled the pulse duration by adjusting the relative timing between the two signals controlling the individual shutters using a digital delay generator. This made adjusting the pulse duration quite simple; we simply added time to the delay to produce correspondingly longer pulses. For this work we define the X-ray peak position as the point halfway between half-maximum crossings and the width as the full separation between the half-maximum crossings of the pulse in the PIN diode signal. We use these definitions (instead of, for instance, the position and width of a Gaussian fit to the data) because pulses with durations longer than ∼20 µs exhibit a distinct ‘flat top’ profile instead of a smooth peak shape owing to the extremely fast opening and closing times of the shutter. We found that, in practice, the pulse position varied by ∼±1 µs while the peak width varied by ∼±0.5 µs from shot to shot.

In earlier experiments we adapted a shutter, originally designed for use with focused laser beams, which uses the voice coil actuator from a computer hard disk drive to move a notched tungsten shutter leaf across the X-ray beam, generating a short pulse (Maguire *et al.*, 2004 ▶; Scholten, 2007 ▶). We drove the shutter using a circuit described by Scholten (2007 ▶). Maguire *et al.* (2004 ▶) demonstrated the potential of this shutter for work with X-rays, and others have adapted this design to shutter a large-aperture X-ray beam with good results (Chua *et al.*, 2010 ▶). Using this shutter while monitoring the X-ray beam intensity with a small ion chamber (JJ X-ray) we were able to produce X-ray pulses shorter than 100 µs with a mechanical delay time which varied by less than 5 µs from shot to shot.

To detect the scattered X-rays we used a MAR 165 X-ray CCD camera (Rayonix, LLC), consisting of a ∼165 mm-diameter active phosphor area coupled to a 2048 × 2048 pixel CCD detector *via* a fiber optic taper. The detector was placed 116 mm downstream from the sample and nominally perpendicular to the incident X-ray beam, with the direct beam blocked by a tungsten beamstop. In this arrangement we recorded ∼15–50% of the diffraction rings (depending on the scattering angle) over a range of scattering vectors from approximately 1 to 5 Å^−1^. For each exposure the CCD camera integrated data over an interval of 2 s, during which the heat-load shutter was open for only 12 ms.

### 4.2. Experimental timing

Timing the experiments at APS was somewhat more complicated than those at CHESS for two reasons. First, although our shutter allowed us to produce very short pulses, the time required to actuate the shutter was relatively long (about 0.5 ms). Second, at CHESS the PAD captured eight frames per experiment, so the timing of these frames relative to each other was known *a priori*; furthermore, they occurred at known times relative to the signal from the photodiode sensing passage of the reaction front (§3.2). At APS, on the other hand, each experiment produced a single diffraction pattern, and the precise timing of the X-ray pulse relative to the position of the front was not known ahead of time. However, using the timing system outlined here we were able to control the relative timing of the X-ray pulse with respect to the passing reaction front to within ∼±100 µs.

Before we discuss the details of the timing scheme, it is useful to outline the sequence of events in the experiment.

(i) Initiate data collection on the CCD camera.

(ii) Open the heat-load (millisecond) shutter.

(iii) Ignite the foil.

(iv) Detect the propagating reaction front.

(v) Actuate the fast (microsecond) shutter, sending a single X-ray pulse through the sample and recording diffraction data with the CCD camera while simultaneously recording pyrometry and PIN diode data on the oscilloscope.

(vi) Close the heat-load shutter.

(vii) Transfer the data from the CCD camera and oscilloscope to a computer.

Each of these processes (with the exception of the CCD camera operation) are tied together by the timing system we designed for these experiments, as described below.

Each data collection event at APS required two manual interventions, the first to initiate the 2 s window of data collection by the CCD camera, and the second to start a computer script that controlled the timing of the remaining events. (The 2 s window was chosen to allow manual initiation of the script while keeping the data collection time low to minimize stray signal collected by the detector.) The computer script sent a signal to the microcontroller in the ignition box (described in §4.4), which then performed the following actions:

(i) Sent the signal to open the heat-load shutter (described in §4.3).

(ii) Waited a user-defined amount of time to allow the heat-load shutter to open.

(iii) Ignited the foil.

Independent electronics opened and closed the heat-load shutter after the predefined (∼12 ms) opening time, as described in §4.3. After ignition of the foil, the light emitted by the advancing reaction front was detected by a fiber-optic-coupled photodiode, in a manner similar to the experiments at CHESS described above. However, to actuate the fast shutter we needed more than ∼0.5 ms advance warning before the reaction front reached the X-ray beam position. So instead of sensing the reaction as it approached the X-ray beam, we positioned the photodiode to collect the light emitted from small ‘detection’ holes located between the ignition pins and the X-ray windows (see §4.4). We collected the photodiode signal on a PC-based oscilloscope (Scope4PC) set to begin data collection when the voltage signal from the photodiode exceeded a predetermined level (∼300 mV). Once the signal from the photodiode exceeded this level, the scope output a voltage level signal some 3–5 ms (depending on the reaction velocity of the particular specimen) before the reaction front crossed the X-ray beam position, giving us sufficient time to actuate the fast shutter. To control the timing of the X-ray pulse with respect to the position of the reaction front, we inserted a delay between the scope trigger output and the shutter input signal using a digital delay and pulse generator (Quantum Composers). Increasing the delay allowed us to probe later times in the reaction progression.

To place the individual diffraction patterns in the correct temporal order, we needed a way to reliably determine the relative timing of the X-ray pulses with respect to the reaction front. Collating the diffraction patterns requires knowledge of when the reaction front crossed the X-ray beam position and the time at which the X-ray pulse occurred, for each specimen. The second point was straightforward: we simply monitored the X-ray pulse intensity using a Si PIN diode (see §4.1) placed downstream of the samples and recorded the signal on an oscilloscope. The first point was somewhat more complicated. To monitor the progress of the reaction at the X-ray beam position we performed *in situ* two-color ratio pyrometry simultaneous to collecting the diffraction data. The pyrometry apparatus and technique are described in §6 below. We collected these data on the same oscilloscope used to collect the PIN diode signal. Analyzing the data after completing the experiment allowed us to determine the timing of the X-ray pulse relative to the moving reaction front to within roughly 10 µs. In this work we define the delay between the reaction front and the X-ray pulse as the difference between the point of maximum curvature in the 1600 nm photodiode signal (not shown) which composes one color in the pyrometry data and the center of the half-maximum crossings of the PIN diode signal.

**Figure 6:**
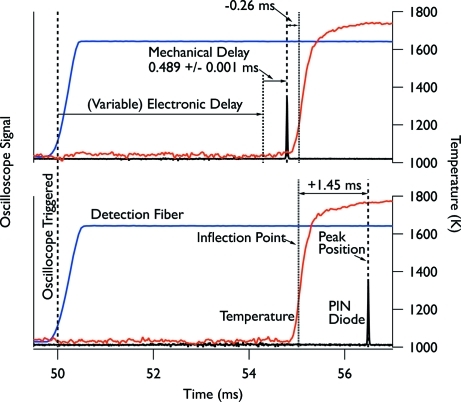
Pyrometry (temperature) (red), PIN diode (black) and detection photodiode (blue) oscilloscope traces for two different delay times when using the fast shutter and CCD camera. The oscilloscope triggers off a rising edge of the detection photodiode signal (∼300 mV). After waiting for a user-selected electronic delay time, the shutter is activated and generates the X-ray pulse, delayed by the (constant) mechanical delay time. The mechanical delay time is very repeatable once the shutter position and voltage reference levels are set; for the conditions used here we measured the mechanical delay time as 0.489 ± 0.001 ms.

Fig. 6 ▶ shows the signals from the PIN diode and detection photodiode overlaid with the pyrometer signal for two different foil exposures. Variations in the signal seen by the detection photodiode can introduce significant errors in the X-ray pulse positioning. However, by fine-tuning the oscilloscope trigger condition and setting it at a repeatable level we could time the X-ray pulse relative to the passing reaction front with good accuracy. Additionally, variations in the reaction front velocity from specimen to specimen also lead to uncertainty in the experimental timing. In practice we observed an RMS jitter in the reaction travel time (over the 12 mm from the detection hole to the pyrometry fiber) of ∼60 µs for foils with reaction velocity of 3.2 m s^−1^. For the two traces in Fig. 6 ▶ the maximum curvature points in the 1600 nm photodiode signals of the pyrometry data line up even closer than this, within 10 µs of each other relative to the oscilloscope trigger condition.

### 4.3. Spatial resolution

For the experiments at APS we focused the X-ray beam using dynamically bent Kirkpatrick–Baez mirrors. The mirrors used for these experiments consist of polished Si mirrors flats coated with a Cr underlayer and a Rh reflective layer, yielding an RMS roughness of 0.7 Å with a maximum slope error of 1.5 µrad. Besides focusing, the mirrors also have the beneficial effect of filtering out the high-energy harmonics of the undulator. Details of the mirror set design and construction can be found elsewhere (Eng *et al.*, 1998 ▶).

We used these mirrors to focus the ‘pink’ X-ray beam (12 keV with 2.3% energy bandpass) directly from the undulator source at sector 7 ID-B at APS to a 7 µm × 6 µm spot at the sample position, roughly 20 cm from the edge of the furthest downstream mirror. The beam size was measured by passing a knife edge through the beam while recording the beam intensity with an ion chamber downstream of the knife edge; the reported beam size is the full width at half-maximum of the approximately Gaussian shape describing the rate of change in intensity as the knife edge transited the beam. We measured the flux in the focused beam using a He-filled ion chamber at ∼3 × 10^13^ photons s^−1^.

Because the mirrors are not water-cooled, we used a water-cooled heat-load shutter upstream of the mirrors to prevent damage to the mirrors and keep the focus stable over the course of the experiment. The shutter consists of two water-cooled copper blocks attached to thick tungsten plates independently moved by two solenoids. A clear plastic box filled with He surrounds the assembly to reduce air scattering and minimize oxidation on the shutter components and upstream beamline windows. This shutter absorbed the heat from the high-power white beam when we were not actively making measurements. The opening and closing of the individual shutter solenoids are handled by a digital delay generator (Stanford Research Systems) which allows adjustment of the opening window time and can be triggered from an external source *via* a TTL voltage level signal. For our experiments we operated with a ∼12 ms opening window, within which we executed our experiment as described in §4.2.

### 4.4. Sample holder and ignition

**Figure 7:**
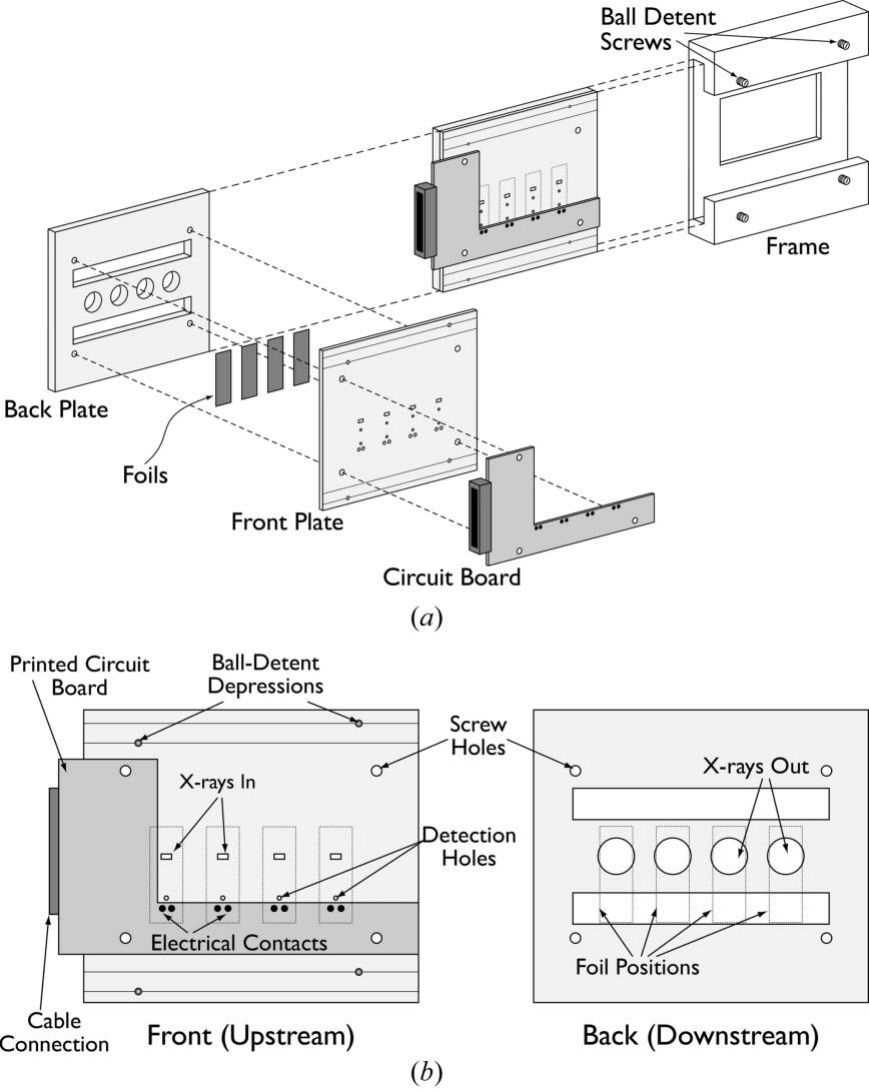
(*a*) Illustration showing the foil-holding cassette used in the experiments performed at APS and the frame it mounts to, along with an exploded view showing how the various parts of the cassette assemble together to hold the foil specimens. The frame attaches to a motorized positioning stage, allowing the samples to be positioned precisely with respect to the X-ray beam. The connector on the left-hand side of the cassette attaches the cassette to the foil ignition box *via* a 15-conductor cable. Ball detents in the frame ensure repeatable positioning of the cassette in the frame. (*b*) Detailed view (not to scale) of the foil-holding cassette. The X-rays enter the upstream side of the cassette through the 0.125 inch × 0.0625 inch rectangular hole, scatter off the foil and exit through the 0.4375 inch circular hole in the downstream side of the cassette.

To mount the specimens for the experiments at APS we used a cassette capable of holding four foils (Fig. 7 ▶) and remotely igniting the individual foil specimens. The earlier experiments at CHESS used a similar but less sophisticated system, which we do not describe here. The cassette consists of two stainless steel plates, between which the foils are clamped. At each foil position the cassette has several holes to allow access to the foil. The small (0.125 inch × 0.0625 inch) rectangular window on the upstream plate and the larger (0.4375 inch-diameter) circular window on the downstream plate provide access for the incident and scattered X-rays, respectively. The large rectangular windows allow the foils to contact spring-loaded electrical pins for ignition and prevent thermal losses due to clamping, which can affect the reaction. The smaller circular holes located between the electrical contacts and the X-ray windows allow optical access to the foils as the reaction propagates from the ignition point to the X-ray beam position. These ‘detection holes’ give an optical signal ∼1–10 ms (depending on the reaction velocity) prior to the reaction front crossing the X-ray beam. This is useful for timing purposes, as described in §4.2. The two plates of the cassette screw together into a single assembly, holding the four foil samples in place for the experiment.

To facilitate quick sample changes, the cassette slides into a frame (Fig. 7*a* ▶) mounted on a motorized *x*/*y* stage allowing horizontal and vertical positioning of the cassette. Ball detent screws in the frame match depressions in the face of the cassette, allowing for repeatable positioning of the cassette in the frame. Each foil is centered in the X-ray beam in its turn, by horizontal translation of the stage and cassette perpendicular to the beam. Once all four samples have been ignited, the cassette can be removed and a new one (with fresh foils) mounted in just a few minutes. The cassette interfaces with the ignition box through a parallel cable which attaches to a custom-printed circuit board *via* a 15-pin connector [visible on the left of Fig. 7(*b*) ▶]. The printed circuit board provides individually addressable connections to the spring-loaded pins in contact with the foil samples.

**Figure 8:**
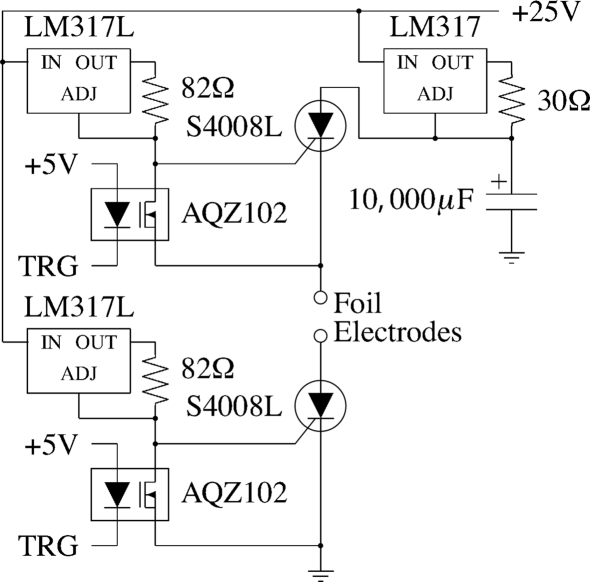
Schematic showing one channel of the ignition circuit in the ignition box used to ignite the foils in the experiments described in the text. TRG is the trigger signal which discharges the capacitor across the foil electrodes, igniting the foil specimen.

To ignite the samples we constructed a capacitive discharge device that can be operated remotely using a computer outside the hutch. The device contains a 10000 µF capacitor which is discharged into one of four channels by S4008L silicon-controlled rectifiers (SCR), as shown in Fig. 8 ▶. We used SCRs for both ignition electrodes (instead of just one) to minimize the risk of cross ignition or multiple ignition of the foils in the cassette. Triggering of the SCRs is managed by a PIC18F4620 microcontroller and AQZ102 optical relays. The microcontroller also supplies a polarity-selectable logic level auxiliary signal for triggering cameras or mechanical shutters. The timing of the experiment is controllable *via* a program from a host computer connected to the microcontroller *via* a serial port. The timing of the various signals is described in §4.2.

## 5. Data comparison

**Figure 9:**
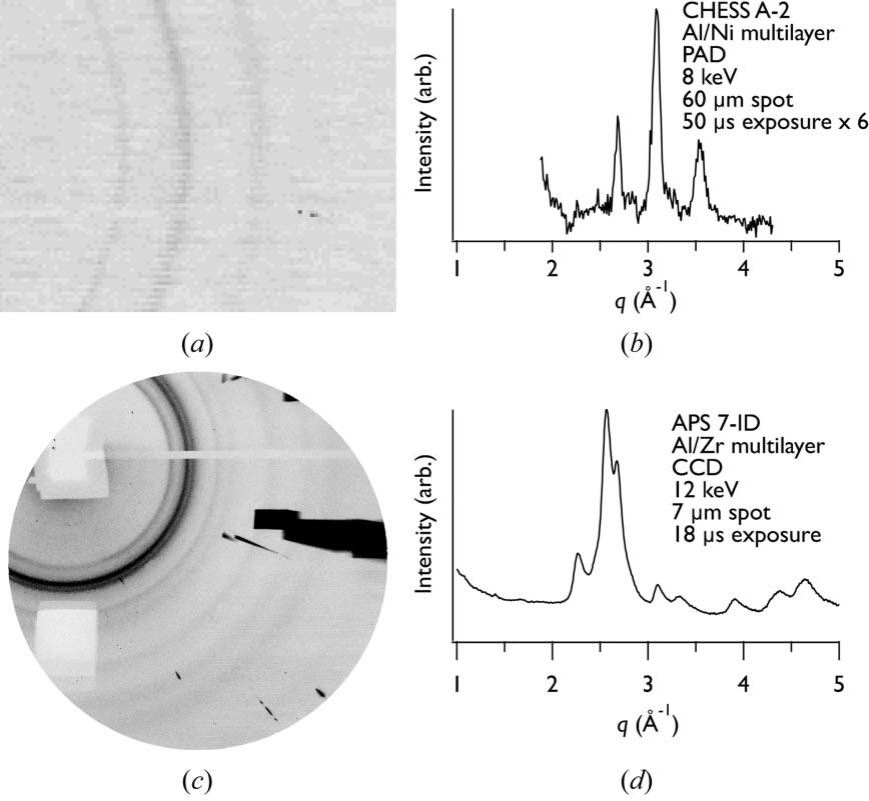
Example data obtained using the two techniques described in this work. (*a*) and (*b*) show, respectively, a detector image after background subtraction and azimuthally integrated data from an unreacted Al_3_Ni_2_ foil using the PAD at CHESS. (*c*) and (*d*) show, respectively, a raw detector image and azimuthally integrated data from an unreacted Al_3_Zr foil using the CCD camera and fast shutter at APS. In (*c*) the pale rectangle and line in the upper portion of the image is the shadow from the tungsten beamstop and the PIN diode arm. The pale rectangle in the lower left portion is the shadow from a lead tape mask placed on the detector face to block a strongly scattering peak from the PIN diode. The dark areas on the right side of the image result from air scattering upstream of the sample cassette. While differences in the experimental details and samples make a direct comparison difficult, the figure shows the improved signal-to-noise ratio, extended *q*-range collected and higher resolution achievable with the combination of the CCD camera and fast shutter at APS compared with the PAD at CHESS. The azimuthally integrated PAD data (*b*) are an average of six separate 50 µs exposures while the CCD data (*d*) are from a single 18 µs exposure. The breadth of the peaks is primarily due to the energy spread (∼2% in both cases) in the incident beams at each source (from the multilayer monochromator used at CHESS and the inherent energy spread in the undulator source at APS). The asymmetric shape of the peaks in the data taken at APS results from the asymmetric energy distribution of the beam from the undulator source at sector 7 at APS (see, for example, Skuza *et al.*, 2007 ▶).

Fig. 9 ▶ shows diffraction data collected at both CHESS and APS. The exposure times are comparable, although the samples and X-ray energies are different. Fig. 9(*a*) ▶ shows a single PAD frame collected at CHESS with a 50 µs integration time (corrected for detector artifacts and with background subtracted). Because the signal-to-noise ratio of a single pattern such as this was not adequate for phase identification purposes, we typically took several (up to ten, depending on the exposure time) such patterns under nominally identical conditions and summed them. For analysis, we azimuthally integrated the summed diffraction patterns to produce one-dimensional patterns, as shown in Fig. 9(*b*) ▶.

The higher flux and larger-format detector at APS yield considerable benefits in terms of signal-to-noise ratio and coverage of reciprocal space. Fig. 9(*c*) ▶ shows a single frame taken with an 18 µs exposure, and the azimuthal average is shown in Fig. 9(*d*) ▶. For the data collected at APS we subtracted a background image from the collected diffraction pattern, corrected for detector artifacts and masked off the area of the detector occluded by the beamstop before azimuthal integration. We used the program *FIT2D* (Hammersley, 1997 ▶, 1998 ▶) to process the APS data.

Comparison of Figs. 9(*b*) and 9(*d*) ▶ shows that the signal-to-noise ratio of the APS data is significantly better than that of the CHESS data. This is the result of both the higher flux at APS and the larger format of the CCD detector (which allows for averaging over a greater fraction of the powder rings). Also, the APS data cover a wider range of scattering vectors, due in part to the larger-format detector but also to the higher X-ray energy.

However, these improvements in the scattering data come at the expense of complicating the experiment. Because only a single frame was captured in each experiment at APS, we had to expend considerable effort in developing suitable techniques for collating the diffraction patterns to place them in the proper temporal sequence. In contrast, the ability of the PAD to collect eight frames in rapid succession from a single specimen eliminates much of the uncertainty in timing. Furthermore, although it was not exploited at CHESS owing to flux limitations, the PAD is capable of shorter exposure times (∼1 µs) than is possible with our fast X-ray shutter (∼20 µs). Table 1 ▶ provides a summary of the specific attributes for each technique.

In principle, one could have the best of both worlds by using both the PAD and a CCD detector simultaneously. In such an experiment the CCD would collect data over a wide *q*-range with an integration time defined by the length of the X-ray pulse produced by the fast shutter (∼20 µs). During this frame the PAD could capture multiple frames in succession, with temporal resolution of ∼3 µs. The PAD data might therefore reveal structural changes occurring too rapidly to be resolved by the CCD, while the larger *q*-range and better signal-to-noise ratio of the CCD data would assist in interpretation of the PAD data. We plan to conduct such experiments in the future. This would be facilitated by a more capable PAD, now in development at Cornell (Koerner *et al.*, 2009 ▶).

## 6. Temperature measurement

Measuring the temperature of the foil as the reaction progresses provides additional information about the phase transformations that are occurring, and, for the experiments performed at APS, makes it possible to collate the diffraction patterns into the proper temporal sequence (§4.2). To do so, we used a fiber-optic-coupled two-color ratio pyrometer similar to that described by Müller & Renz (2001 ▶). By using two-color ratio pyrometry we minimized the dependence of the temperature measurement on the (likely rapidly changing) surface emissivity of the foil specimen itself. While surface conditions (emissivities) undoubtedly change with phase transformations and temperature, the ratio pyrometer signal depends not on the absolute emissivities but only on the ratio of emissivity at 1400 nm to that at 1600 nm. This allowed us to monitor the temperature of the foils in real time as the reaction progressed without having to make assumptions about how the absolute emissivity changed during the transformation.

In the pyrometer the light from the reaction front is collected with an optical fiber and split into two paths, leading to dichroic filters that pass different ranges of wavelengths [Spectrogon NB-1395 (1395 ± 15 nm) and NB-1600 (1600 ± 15 nm)]. The intensity of light in each wavelength band is measured with separate photodiodes [Thorlabs PDA10CS (1395 nm) and PDA400 (1600 nm)], the output of which is recorded with an oscilloscope (LeCroy or Custom Engineering Design). By taking the ratio of the two photodiode signals and applying suitable calibrations (Trenkle, 2008 ▶) we can determine the corresponding temperature during the reaction. We used 200 µm-diameter optical fibers with a numerical aperture of 0.22 (Thorlabs) in the pyrometer. This resulted in a collection spot of 0.5–1 mm diameter, much larger than the X-ray spot size. Temperature measurements, therefore, are averages over this spot size, though biased towards higher temperatures since the light intensity scales as *I* ≃ *T* ^4^. In this experiment we estimate errors in the measured temperature of ±225 K owing to the uncertainty in spatial displacement between the center of the X-ray beam and the center of the pyrometer sampling area. For the experiments at APS, pyrometry data were recorded simultaneously with the diffraction data. We did not perform pyrometry at CHESS; for those experiments, pyrometry data was recorded separately, on nominally identical foils, as described elsewhere (Trenkle, 2008 ▶; Trenkle *et al.*, 2010 ▶).

## 7. Implications and conclusions

The results shown above demonstrate the ability to make X-ray diffraction measurements with temporal resolution on the microsecond scale and spatial resolution of the order of a few micrometers. While these resolutions are orders of magnitude larger than those possible with a pump–probe approach (see, for instance, Grigoriev *et al.*, 2006 ▶), multi-shot pump–probe techniques are only applicable to reversible transformations that can be repeatedly excited. Our technique, in contrast, is suitable for studying irreversible structural changes in materials, especially those that occur with significant timing jitter with respect to an external stimulus.

While our efforts to date have focused solely on self-propagating reactions in metallic multilayers, it is worth considering what other processes in materials occur on similar time and length scales and which might therefore be amenable to study with these techniques. One example, closely related to the self-propagating formation reactions described above, is explosive crystallization of amorphous thin films, which is believed to be mediated by the formation of a liquid phase at the crystalline/amorphous interface (Leamy *et al.*, 1981 ▶). More generally, crystal/melt interface velocities of the order of 10 m s^−1^ are common, and both melting and freezing might be profitably studied with these techniques. A related area would be crystallization of polymers, which has already been studied with time-resolved X-ray scattering as well as X-ray microdiffraction (Hughes *et al.*, 1999 ▶; Riekel, 2003 ▶). Furthermore, while arc welding has been successfully studied using time-resolved X-ray microdiffraction on the scale of a few hundred milliseconds (Elmer *et al.*, 2008 ▶), the enhanced resolutions of this technique may enable studies of the faster heating and cooling rates and more localized nature of laser or electron beam microwelding (He *et al.*, 2005 ▶).

Another area of application could be mechanical deformation of materials. For instance, one could study the evolution of structure in response to cyclic loading. This has been done for fatigue loading, probing a relatively large volume of material (see, for example, Park *et al.*, 2007 ▶), but with the spatial resolution of the techniques described here, one could imagine studying localized regions near the tip of a fatigue crack. One concern with small X-ray beam sizes is that if the grain size of the material is large, the small volume of material probed may not contain a statistically representative sample of crystals. In this regard the use of pink beam is an advantage, because of the relatively broad distribution of wavelengths.

In considering other applications of the techniques described here, one point to keep in mind is the need for a suitable signal for triggering the X-ray pulse (for single-shot experiments with a slow detector) or for initiating the detector collection (for the multiple-shot PAD experiments). The self-propagating reactions studied here are conveniently bright, allowing the light emitted from the reaction front to be used for triggering.

In conclusion, we have presented two techniques for performing time-resolved X-ray microdiffraction on self-propagating high-temperature synthesis reactions in metal multilayer foils. In our first approach we used capillary optics to produce a focused X-ray beam and a fast pixel array detector. With this combination we achieved spatial resolution of 60 µm and temporal resolution of 55 µs (the latter limited by the beamline flux rather than the detector). In our second technique we focused the X-rays with Kirkpatrick–Baez mirrors and generated temporal resolution by using a fast X-ray shutter, recording the data with a relatively slow CCD camera. This gave us spatial resolution <10 µm and temporal resolution <20 µs. Use of the larger-format CCD camera has the advantages of providing better signal-to-noise ratio and covering a larger range of reciprocal space, but comes at the cost of significant experimental complication. In particular, because only one measurement can be made per sample, the need arises for accurate experimental timing to allow collation of the diffraction patterns from multiple specimens into the proper sequence. We expect that the techniques described here could be profitably applied to the study of many irreversible transformations in materials that have characteristic length scales of the order of micrometers and which evolve over sub-millisecond time scales.

## Figures and Tables

**Table 1:** Comparison of the two sets of experiments described here

	APS/shutter/CCD	CHESS/PAD
Focusing	KB mirrors	Capillary optics
Spatial resolution	∼7 µm	∼60 µm
Shuttering	Mechanical shutter	Fast detector collection
Temporal resolution	∼18 µs	55 µs (flux limited)
Detector	MAR 165 CCD camera	High-speed pixel array detector
Pixels	2048 × 2048 pixels	100 × 92 pixels
Pixel size	80 µm	150 µm
Activation time	∼500 µs	0.05 µs
Patterns/specimen	1	8
Signal-to-noise	Good	Requires averaging
